# Homotypic CARD-CARD interaction is critical for the activation of NLRP1 inflammasome

**DOI:** 10.1038/s41419-020-03342-8

**Published:** 2021-01-11

**Authors:** Zhihao Xu, Ying Zhou, Muziying Liu, Huan Ma, Liangqi Sun, Ayesha Zahid, Yulei Chen, Rongbin Zhou, Minjie Cao, Dabao Wu, Weidong Zhao, Bofeng Li, Tengchuan Jin

**Affiliations:** 1 Department of Obstetrics and Gynecology, The First Affiliated Hospital of USTC, Division of Life Sciences and Medicine, University of Science and Technology of China, Hefei, Anhui 230001 China; 2 Hefei National Laboratory for Physical Sciences at Microscale, The CAS Key Laboratory of Innate Immunity and Chronic Disease, School of Basic Medicine Sciences, Division of Life Sciences and Medicine, University of Science and Technology of China, Hefei, Anhui 230027 China; 3 College of Food and Biological Engineering, Jimei University, Xiamen, Fujian 361021 China; 4 CAS Center for Excellence in Molecular Cell Science, Chinese Academy of Science, Shanghai, 200031 China; 5 Department of Medical Oncology, The First Affiliated Hospital of USTC, Division of Life Sciences and Medicine, University of Science and Technology of China, Hefei, Anhui China 230001

**Keywords:** Molecular biology, X-ray crystallography, Autoimmune diseases

## Abstract

Cytosolic inflammasomes are supramolecular complexes that are formed in response to intracellular pathogens and danger signals. However, as to date, the detailed description of a homotypic caspase recruitment domain (CARD) interaction between NLRP1 and ASC has not been presented. We found the CARD–CARD interaction between purified NLRP1^CARD^ and ASC^CARD^ experimentally and the filamentous supramolecular complex formation in an in vitro proteins solution. Moreover, we determined a high-resolution crystal structure of the death domain fold of the human ASC^CARD^. Mutational and structural analysis revealed three conserved interfaces of the death domain superfamily (Type I, II, and III), which mediate the assembly of the NLRP1^CARD^/ASC^CARD^ complex. In addition, we validated the role of the three major interfaces of CARDs in assembly and activation of NLRP1 inflammasome in vitro. Our findings suggest a Mosaic model of homotypic CARD interactions for the activation of NLRP1 inflammasome. The Mosaic model provides insights into the mechanisms of inflammasome assembly and signal transduction amplification.

## Introduction

Vertebrates have evolved an innate immune system as first line of defense against pathogen-derived molecules. The system includes intracellular pathogen-associated molecular patterns (PAMPs) and danger-associated molecular patterns (DAMPs)^1^. Pattern recognition receptors (PRRs) recognize various microbial structures and activities that initiate the innate immune response of cells. PRRs consist of many protein families, including Toll-like receptors, NOD-like receptors (NLRs), RIG-I-like receptors, AIM2-like receptors, and C-type lectin receptors^2,3^. Formation of a multi-protein complex known as the inflammasome initiates a strong inflammatory response caused by the activation of the cysteine proteases caspase-1 and caspase-4/5/11^4^. Inflammasomes are categorized as NLRP1, NLRP3, NLRC4, or AIM2 inflammasomes, depending on the type of sensor protein^5^. Malfunction of inflammasomes is associated with numerous autoimmune disorders, including vitiligo, Type II diabetes, and celiac disease^6,7^.

Inflammasomes are studied to better understand the cellular danger signal monitoring and signal transduction mechanisms^8^. NLRP1, a member of the nucleotide-binding-domain leucine-rich-repeat (NLR) superfamily^9^, was the first NLR discovered that forms inflammasome in the innate immune system^10^. NLRP1 senses danger signals and performs self-cleavage to release its C-terminal segment (UPA-CARD domains), whcich initiates downstream signal transduction pathways. Self- and homotypic interactions of ASC (apoptosis-associated speck-like protein containing a CARD) can mediate the assembly of a supramolecular complex, ultimately activating caspase-1 by an unknown mechanism^11^. The activated caspase-1 cleaves specific cytokines (interleukin-1β and -18) and Gasdermin D, which induces pro-inflammatory cell death known as pyroptosis^12,13^.

NLRP1 is a unique member of the NLRP family, because it has an extra FIIND and CARD following the Leucine-rich repeat domain in the C terminus. Furthermore, a PYD does not appear to be critical for the function because of the lack of a PYD as found in the N-terminal of murine homolog^14^. Unlike human NLRP1, there are three paralogs of *Nlrp1*(*Nlrp1a*,*b*,*c*) in mouse genome, and murine NLRP1A and NLRP1B inflammasome can be assembled by recruitment of caspase-1 either with ASC or without ASC^15–17^. Recently, a novel activation mechanism, called the functional degradation model, of the murine NLRP1B inflammasome was proposed^18,19^. In the model, the C-terminal function-to-find domain (FIIND) of NLRP1B self-cleaves to initiate the function of the NLRP1B inflammasome. The function of inflammasome is enabled by the degradation of the N terminus of NLRP1B by a lethal factor protease to expose a destabilizing N-degron. The inflammasome releases its C-terminal fragment that consists of a partial FIIND domain and an entire CARD domain, to recruit caspase-1 to the inflammasome for caspase activation. Interestingly, inhibitors of dipeptidyl peptidases DPP8 and DPP9 were also found to activate the NLRP1B inflammasome by an endogenous degradation pathway. It is unknown how DPP8/9-mediated signaling pathways regulate the auto-inhibited or activated state of NLRP1B^20,21^. The activation mechanism of human NLRP1, which consists of an extra N-terminal PYD domain different from murine NLRP1B, remains unknown^18,22^. Mutations of its PYD domain are related to several skin diseases, indicating an important function of PYD in the mechanism of human NLRP1 activation^23^. More investigations are needed to unravel the complicated regulation mechanisms of human NLRP1 in innate immunity.

NLRP1 is also an adapter protein, which along with ASC bind to themselves or each other by homotypic interactions among the death domain (DD) contained within them (e.g., PYD, CARD)^24^. The members of the DD superfamily engage in assembly of filaments by three different interaction types: Type I, II, and III. The binding interfaces do not overlap so that a protein with more than one type can interact with itself or other DD-containing proteins simultaneously. CARD–CARD interactions, such as pro-caspase-9/Apaf-1 and CED-4/CED-9 complexes, have been characterized by structural studies^25,26^. However, little information is available about CARD–CARD interactions involved in the assembly of NLRP1 inflammasome. Our understanding of the molecular details of the interaction between NLRP1^CARD^ and ASC^CARD^ is limited.

We found that the CARD of NLRP1 (NLRP1^CARD^) and the CARD of ASC (ASC^CARD^) interact with each other in solution and cells. We investigated the function of the CARDs of each protein and found that NLRP1^CARD^ binds with ASC^CARD^ to form filament complexes using each of the three DD interaction types. Heteromeric and homomeric interactions play significant roles in inflammasome activation and danger signal transduction amplification in NLRP1. The assembly of NLRP1^CARD^ and ASC^CARD^ for signal transduction amplification was named as Mosaic model. The mutations of three asymmetric interactions between NLRP1^CARD^ and ASC^CARD^ led to the suppression of NLRP1 inflammasome activation. We propose a ASC-dependent activation model-the Mosaic model-that reveals new insights into the formation of NLRP1 inflammasome and highlights the importance of CARDs in the mechanism of NLRP1 signal transduction amplification.

## Results

### NLRP1 binds with ASC by homotypic CARD–CARD interaction

The self-cleavage of NLRP1 and release of the C-terminal fragment containing the partial FIIND and the entire CARD are necessary for NLRP1 activation. The C-terminal fragment subsequently recruits ASC to activate caspase-1^27^. This function differs from NLRP3 and AIM2 inflammasomes whose N-terminal PYD domains recruit adapter ASC to form the fiber-like assembly^28,29^. We first wanted to investigate the role of CARD–CARD interactions in the human NLRP1 inflammasome.

**Fig. 1:**
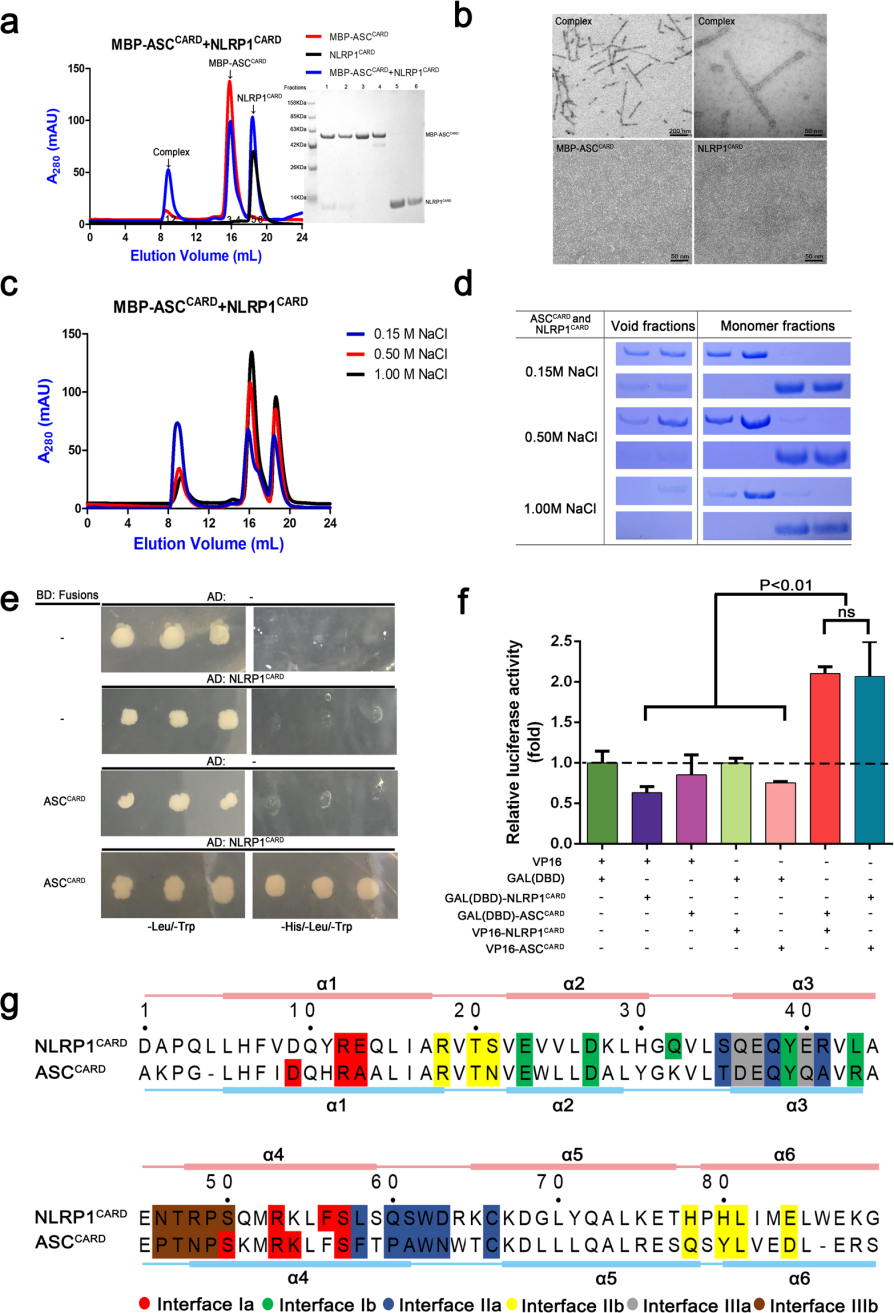
NLRP1^CARD^ interacts with ASC^CARD^ by homotypic CARD–CARD interaction in solution and cells. **a** Size-exclusion chromatograph of the MBP-ASC^CARD^/NLRP1^CARD^ complex. ASC^CARD^ was fused with an N-terminal His-tag and incubated with untagged NLRP1^CARD^, which was first purified by Ni-affinity chromatography. The complex eluted in the void position on a Superdex^TM^ 24 gel filtration column. **b** A negative-stain EM image of MBP-ASC^CARD^/NLRP1^CARD^ complex. **c** Size-exclusion chromatograph of the MBP-ASC^CARD^/NLRP1^CARD^ complex in different salt concentrations. **d** High salt significantly disrupted filament formation. **e** Y2H analysis of the NLRP1^CARD^ and ASC^CARD^ interaction. Yeast cells co-expressing GAL4 DNA-binding domain (BD)-ASC ^CARD^ fusion and GAL4 activation domain (AD)-NLRP1^CARD^ fusion were grown on agar plates lacking leucine and tryptophan (-Leu/-Trp) for transformant growth and lacking histidine, leucine, and tryptophan (-His/-Leu/-Trp) for detecting CARD–CARD interaction. Three individual clones for each combination were plated. (–) denotes empty vector control. **f** Measurement of the protein–protein interaction of wild-type NLRP1^CARD^ with ASC^CARD^ by the M2H experiment. Luciferase activity in the HEK293T cells was normalized to *Renilla* and data were presented as the fold of negative control. Mean values ± SEM are representative of three independent experiments. **g** Structure-based sequence alignment of NLRP1^CARD^ (NP_127497.1) and ASC^CARD^ (NP_037390.2). Different colors are highlighted to show the interfacial residues involved in the three asymmetric interactions of death domain superfamily. The secondary structure of NLRP1^CARD^ and ASC^CARD^ are labeled on the top and bottom, respectively.

We evaluated the complex formation of NLRP1^CARD^ and ASC^CARD^ in an in vitro proteins solution. ASC^CARD^ formed homotypic aggregates and precipitated from the solution. To overcome the precipitation problem, the maltose-binding protein which is shown to be a crystallization chaperone^30^ was linked to ASC^CARD^ (MBP-ASC^CARD^). Most of the MBP-ASC^CARD^ eluted as a single symmetrical peak at approximately 16 mL from the size-exclusion chromatographic column, indicating that the MBP-ASC^CARD^ was homogenous and existed as a monomer in solution. After incubating MBP-ASC^CARD^ with a threefold concentration of NLRP1^CARD^ for 4 h, an MBP-ASC^CARD^/NLRP1^CARD^ complex was observed by size-exclusion chromatography (Fig. 1a). The MBP-ASC^CARD^/NLRP1^CARD^ complex eluted at ~9 mL, which corresponded to a molecular weight of ~400 kDa. By the gray scales analysis of the polyacrylamide gel electrophoresis (PAGE) gel, the molecular ratio between NLRP1^CARD^ and ASC^CARD^ is ~1 : 2 in the gel filtration assay (Supplementary Fig. S1a). The analysis of the negative-stain EM showed that the MBP-ASC^CARD^/NLRP1^CARD^ complex formed extended filament structures (Fig. 1b). The MBP fusion tag did not appear to interfere with the interaction between NLRP1^CARD^ and ASC^CARD^, because there was a long flexible linker between MBP and ASC^CARD^. The filaments formed from this complex had varying lengths but were uniform in the diameter (~10 nm), which is consistent with the cryogenic electron microscopy (cryo-EM) structure of ASC^CARD^ and NLRC4^CARD^-only filaments^31^. We performed size-exclusion chromatography analysis at different concentrations of salt, to investigate the importance of charge complementary in the NLRP1^CARD^ and ASC^CARD^ interaction. The MBP-ASC^CARD^/NLRP1^CARD^ complex showed salt dependence at 0.15, 0.5, and 1.0 M NaCl using gel filtration analysis. The MBP-ASC^CARD^/NLRP1^CARD^ complex decreased and the monomer peaks increased with increasing NaCl concentration. This indicates that ionic interactions play an important role in NLRP1^CARD^ and ASC^CARD^ interactions (Fig. 1c, d). We conclude that charge complementary is essential in mediating NLRP1^CARD^/ASC^CARD^ filament formation.

We examined the interaction between NLRP1^CARD^ and ASC^CARD^ in cells using yeast two-hybrid (Y2H) and mammalian two-hybrid (M2H) systems. A strong binding interaction was verified between wild-type full-length NLRP1 and ASC in the M2H system. However, there was no interaction between CARD-deletion mutants of NLRP1 and ASC, implying that NLRP1 recruits ASC through CARD domains (Supplementary Fig. S1b). Yeast cells expressing both NLRP1^CARD^ and ASC^CARD^ grew on SD/-Trp/-Leu/-His media, indicating that the NLRP1^CARD^ and ASC^CARD^ interact with each other directly (Fig. 1e). We observed that, in the M2H system, the interaction between NLRP1^CARD^ and ASC^CARD^ was significantly stronger than in the control groups (Fig. 1f). Immunoblotting experiments showed that NLRP1^CARD^ and ASC^CARD^ were expressed at similar levels in the M2H system (Supplementary Fig. S1c). We conclude that homotypic CARD–CARD interactions mediate NLRP1^CARD^/ASC^CARD^ complex formation in solution and the recruitment of ASC by NLRP1 in cells.

Primary sequences of NLRP1^CARD^ and ASC^CARD^ were more conserved than other CARD proteins by sequence alignment of nine kinds of CARD proteins (Supplementary Fig. S2). According to charged residues and three-dimensional position in the NLRP1^CARD^ and ASC^CARD^ structure, the interfacial residues of the three major asymmetric interfaces and secondary structures in NLRP1^CARD^ and ASC^CARD^ showed a highly similarity, perhaps indicating a more compatible protein–protein interaction between NLRP1^CARD^ and ASC^CARD^ (Fig. 1g). The sequence and structural similarity between NLRP1^CARD^ and ASC^CARD^ may provide a foundation for NLRP1 and ASC to form heterotypic supramolecular complexes via their CARD domains during NLRP1 inflammasome activation.

### AI of NLRP1^CARD^ is important for binding with ASC^CARD^

**Fig. 2:**
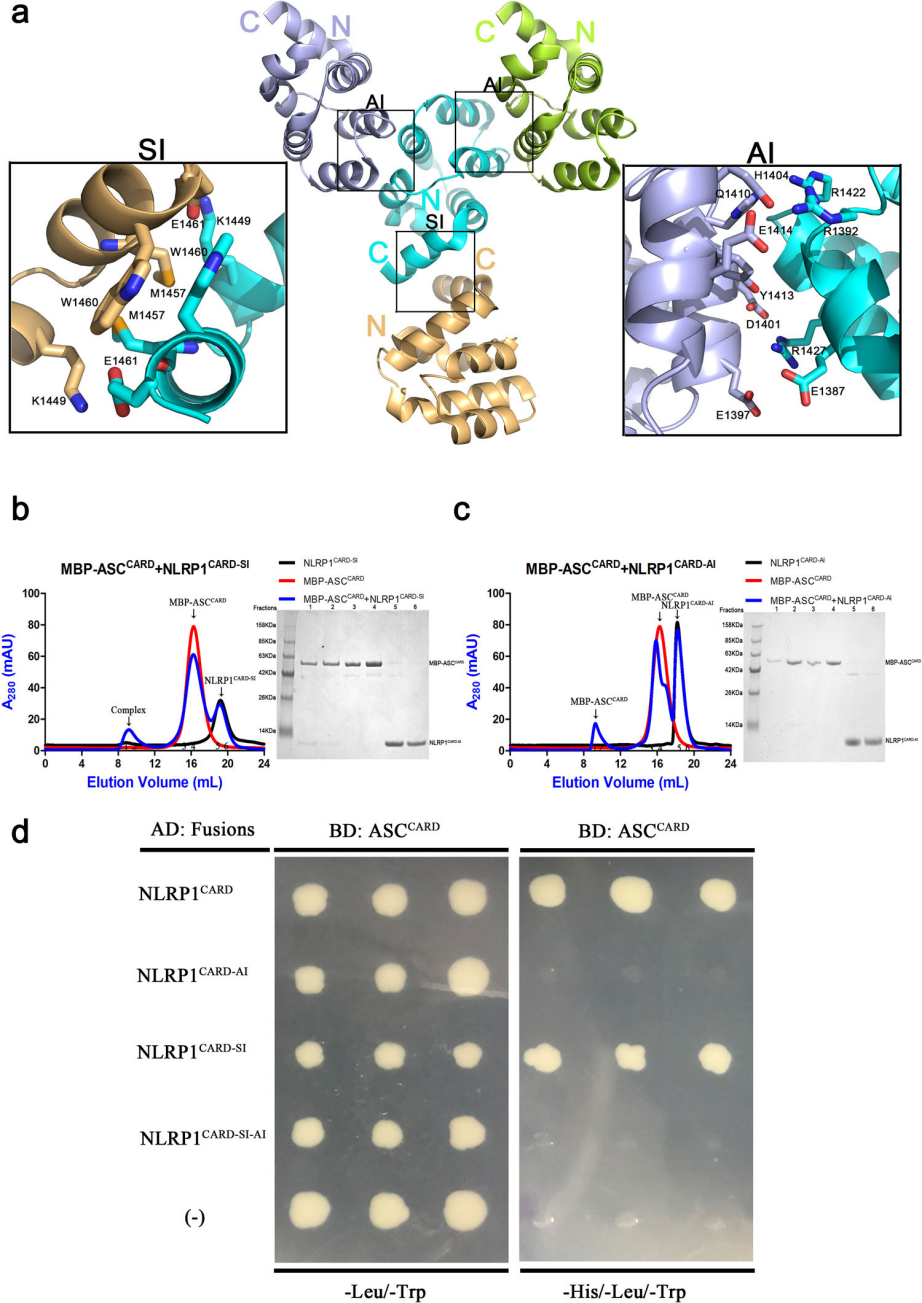
NLRP1^CARD^ and ASC^CARD^ interaction depends on the asymmetric interface of NLRP1^CARD^. **a** CARD–CARD symmetric interaction (SI) and asymmetric interaction (AI) interfaces of NLRP1^CARD^ in the crystal lattice. The acidic residues are labeled in black. **b** Size-exclusion chromatograph of the MBP-ASC^CARD^/NLRP1^CARD-SI^ complex. ASC^CARD^ was fused with an N-terminal His-tag and incubated with untagged NLRP1^CARD-SI^, which was first purified by Ni-affinity chromatography. The complex eluted in the void position on a Superdex^TM^ 24 gel filtration column. **c** Size-exclusion chromatograph of the mixture of MBP-ASC^CARD^ and NLRP1^CARD-AI^. ASC^CARD^ was fused with an N-terminal His-tag and incubated with untagged NLRP1^CARD-AI^, which was first purified by Ni-affinity chromatography. The protein eluted in the less aggregated position on a Superdex^TM^ 24 gel filtration column. **d** Y2H analysis of the interaction between ASC^CARD^ and NLRP1^CARD^ mutations. Three individual clones for each combination were plated. (−) denotes empty vector control.

X-ray crystallography of the CARD–CARD interactions between NLRP1^CARD^ proteins found that NLRP1^CARD^ crystals formed two kinds of lattice contacts^22^. In one lattice, a symmetric interface (SI) was formed between K1449, M1457, W1460, and E1461 from the sixth α-helices of NLRP1^CARD^ dimers. In the second lattice, an asymmetric interface (AI) was formed between E1397, D1401, and E1414 from helices 2 and 3 of one NLRP1^CARD^, and among R1392, R1422, and R1427 from helices 1 and 4 of a second NLRP1^CARD^ (Fig. 2a). Analysis of the interface area and bonds of the SI and AI interfaces using the Protein Interfaces, Surface, and Assemblies (PISA) server^32^ showed that SI and AI have interface areas of 344.4 and 367.7 Å^2^, respectively. The AI interface had 7 hydrogen bonds and 6 salt bonds more than SI interface. A close examination of the SI and AI structures revealed that the hydrophobic residues M1457 and W1460 are heavily involved in the SI interface, whereas ionic interactions are more dominant in the AI interface.

We generated structure-guided mutants to investigate the effects of mutations on the interactions of the two interfaces. An NLRP1^CARD-SI^ mutant contained mutations in the helix 6 residues: K1449A, M1457A, W1460A, and E1461A. The NLRP1^CARD-AI^ mutant contained mutations in helices 1 and 4 residues: R1392A, R1422A, and R1427A. The NLRP1^CARD-SI-AI^ mutant contained both sets of mutations (Fig. 2a and Supplementary Fig. S3a). Circular dichroism (CD) spectroscopy of NLRP1^CARD-SI^ (K1449A, M1457A, W1460A, and E1461A), NLRP1^CARD-AI^ (R1392A, R1422A, and R1427A), and NLRP1^CARD-SI-AI^ (K1449A, M1457A, W1460A, E1461A, R1392A, R1422A, and R1427A) showed the same negative peaks between 200 and 240 nm as NLRP1^CARD^, indicating that NLRP1^CARD-SI^, NLRP1^CARD-AI^, NLRP1^CARD-SI-AI^, and NLRP1^CARD^ have the similar secondary structure in agreement with the observed high α-helical content of DD superfamily members (Supplementary Fig. S3b). Thermal denaturation CD spectrum analysis demonstrated that the mutant proteins had similar thermal stabilities (Supplementary Fig. S3c). Size-exclusion chromatography coupled with multi-angle static light scattering (MALS) of NLRP1^CARD^ mutants demonstrated that they were predominantly monomeric. However, in the same analysis of NLRP1^CARD^ displayed a pattern of monomer-to-dimer equilibrium (Supplementary Fig. S4a–d). These results indicate that both SI and AI interfaces contribute to the homotypic interaction of NLRP1^CARD^ in solution.

We investigated whether the CARD–CARD interaction between NLRP1^CARD^ and ASC^CARD^ involves the SI or the AI interface of the NLRP1^CARD^. Incubation of the NLRP1^CARD-SI^ with MBP-ASC^CARD^ resulted in complex formation (Figs. 2b and 1a), whereas incubation of the NLRP1^CARD-AI^ with MBP-ASC^CARD^ did not result in supramolecular complexes (Figs. 2c and 1a). We used CARD9^CARD^ as a control to test the specificity of the NLRP1^CARD-SI^ and ASC^CARD^ interaction. We found that NLRP1^CARD-SI^ interacted with ASC^CARD^, but not with other CARD-containing proteins (Supplementary Fig. S5a, b). Y2H assays and size-exclusion chromatography both demonstrated that NLRP1^CARD-SI^, instead of NLRP1^CARD-AI^ or NLRP1^CARD-SI-AI^, interacted with ASC^CARD^ (Fig. 2d). The interaction between NLRP1^CARD^ and ASC^CARD^ requires the AI from NLRP1.

### The structure of the ASC^CARD^ is highly conserved

Results from structural studies of ASC^CARD^ were used to determine the contribution of ASC^CARD^-specific residues to the formation of filaments with NLRP1^CARD^. An investigation of different linkers between MBP and ASC^CARD^ found a linker that allows the crystallization of MBP-ASC^CARD^. Supplementary Table S1 shows the crystallographic data collection, model building, and refinement statistics.

**Fig. 3:**
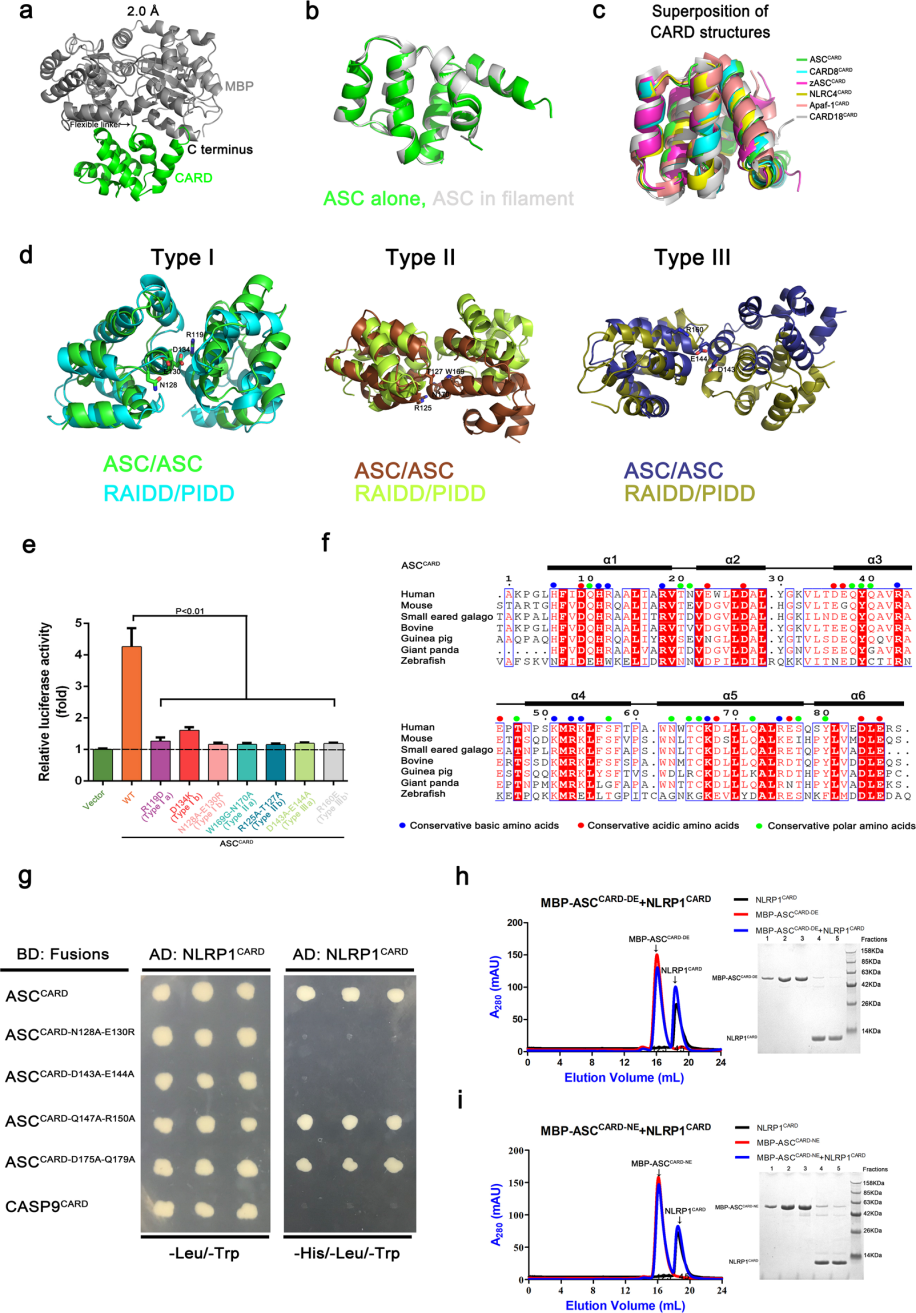
Critical sites on ASC^CARD^ surface for interacting with NLRP1^CARD^. **a** Crystal structure of the CARD of ASC (green) with a MBP tag (gray) at 2.0 Å resolution. **b** Comparison of the initial ASC^CARD^ subunit model (green) and the subunit structure after refinement against the cryo-EM density (gray, PDB ID: 6N1H). **c** Superposition of all six known CARD structures, showing the highly conserved CARD structure. **d** Superposition of the ASC/ASC dimers with RAIDD/PIDD dimers in the Type I, II, and III interactions. **e** Measurement of the self-interaction of wild-type ASC^CARD^ by the M2H experiment. Luciferase activity in the HEK293T cells was normalized to *Renilla* and data were presented as the fold of negative control. Mean values ± SEM are representative of three independent experiments. **f** Structure-based sequence analysis of the ASC^CARD^ protein. Amino acid position is indicated on the top. GenBank accession numbers for ASC^CARD^: Human (NP_037390.2); Mouse (NP_075747.3); Small-eared-galago (XP_003795885.1); Bovine (NP_777155.1); Guinea pig (XP_003478302.1); Giant panda (XP_002924797.1); and Zebrafish (NP_571570.2). **g** Mapping important ASC^CARD^ surface sites for interaction with NLRP1^CARD^ by Y2H analysis. Three individual clones for each combination were plated. **h** Size-exclusion chromatograph of the mixture of MBP-ASC^CARD-DE^ and NLRP1^CARD^. ASC^CARD-DE^ was fused with an N-terminal His-tag and incubated with untagged NLRP1^CARD^, which was first purified by Ni-affinity chromatography. The protein eluted in the less aggregated position on a Superdex^TM^ 24 gel filtration column. **i** Size-exclusion chromatograph of the mixture of MBP-ASC^CARD-NE^ and NLRP1^CARD^. ASC^CARD-NE^ was fused with an N-terminal His-tag and incubated with untagged NLRP1^CARD^, which was first purified by Ni-affinity chromatography. The protein eluted in the less aggregated position on a Superdex^TM^ 24 gel filtration column.

The crystallographic structure of human ASC^CARD^ is composed of six amphipathic α-helices engaging a hydrophobic core, similar to what has been found for other CARD domains^25,33,34^. The structure was refined to 2.0 Å resolution and exhibits excellent agreement with the cryo-EM structure of ASC^CARD^ in filaments with an root mean squared deviation (RMSD) of 0.502 Å^31^ (Fig. 3a, b). The crystal structure confirmed that the structure of ASC^CARD^ is not affected by the MBP fusion tag (Fig. 3a). The structure of the CARD in ASC^CARD^ shares similar structural characteristics with other DD superfamily members. The interruption in helix 1 of ASC^CARD^ showed that the native conformation is relatively conserved. Superposition of the ASC^CARD^ structure with structures of CARD subfamily members, including CARD8^CARD^ (4IKM), zASC^CARD^ (5GPQ), NLRC4^CARD^ (6N1I), Apaf-1^CARD^ (1CY5), and CARD18^CARD^ (1DGN), with RMSD values <1.4 Å confirmed that the three-dimensional structure with six anti-parallel α-helices folded in a Greek key arrangement is a highly conserved feature of a DD fold (Fig. 3c).

### Identification of homotypic CARD interface of ASC^CARD^

The functions of DD proteins often require homotypic DD interactions^24^. It has been shown that filament assembly by members of the DD superfamily is mediated by the three major asymmetric interfaces^35^. Each of these three asymmetric interactions were observed in the cryo-EM ASC^CARD^ filament structure^31^.

We aligned the three major asymmetric interfaces of the DD superfamily responsible for filament formation (Type I, II, and III) with the corresponding interfaces in the PIDD/RAIDD DD complex (Fig. 3d). We found that the Type I interaction mediated by the residues from helices 1 and 4 (Type Ia) of ASC^CARD^ and residues from helix 2 and 3 (Type Ib) of a second ASC^CARD^ is highly conserved. The Type II interaction formed by the residues in the loop between helix 4 and helix 5 (Type IIa) of ASC^CARD^ and residues at the helix 5–helix 6 corner (Type IIb) of a second ASC^CARD^ is less conserved. The Type III interaction formed by residues at helix 3 (Type IIIa) of one ASC^CARD^ and residues at helix 3–helix 4 corner (Type IIIb) of a second ASC^CARD^ is also less conserved.

The M2H system was employed to validate the functional relevance of interfacial residues for the self-interaction of ASC^CARD^ determined by the structure analysis. ASC^CARD^ demonstrated a strong interaction with itself when expressed in HEK293T cells (Fig. 3e). According to the conserved, charged residues, three-dimensional position in the MBP-ASC^CARD^ structure, and the published data^31,36^, some residues were picked to test the self-interaction of ASC^CARD^. Mutations of the Type I residues R119D, D134K, and N128A/E130R considerably decreased ASC^CARD^ self-interaction. Concomitant mutations of the Type IIa residues W169G/N170A and the Type IIb residues R125A/T127A abolished ASC^CARD^ self-interaction. Mutations of the Type III residues R160E and D134A/E144A also decreased ASC^CARD^ self-interaction. These in vitro mutagenesis studies show that Type I, II, and III interfaces are involved in the self-interaction of ASC^CARD^.

### Critical ASC^CARD^ surface sites for interaction with NLRP1^CARD^

We used the Y2H system to map critical surface sites mediating NLRP1^CARD^ and ASC^CARD^ interaction to understand the molecular interaction in NLRP1 inflammasome assembly. Sequence alignment of ASC^CARD^ from different species was performed to identify the conserved amino acids that may participate in the interactions between NLRP1^CARD^ and ASC^CARD^ (Fig. 3f). Most of the conserved residues are either hydrophobic amino acids involved in the packing of hydrophobic cores or charged interfacial amino acids interacting with other proteins. Some conserved charged residues (N128, E130, D143, E144, Q147, R150, D175, and Q179) were identified as possibly involved in the interaction between NLRP1^CARD^ and ASC^CARD^. Site-directed mutagenesis on the Y2H construct of ASC^CARD^ showed that only the double mutants of D143A/E144A and N128A/E130R grew on SD/-Trp/-Leu/ plates instead of SD/-Trp/-Leu/-His plates, demonstrating the roles of D143, E144, N128, and E130 in the interaction between NLRP1^CARD^ and ASC^CARD^ (Fig. 3g). Mutations of these four conserved charged residues abrogated the interaction between NLRP1^CARD^ and ASC^CARD^. We tested the interaction between NLRP1^CARD^ and CASP9^CARD^, a non-related CARD domain, to confirm the specificity of the CARD–CARD interactions between NLRP1^CARD^ and ASC^CARD^. NLRP1^CARD^ and human-derived CASP9^CARD^ did not interact with each other in the Y2H system, demonstrating the specificity of CARD–CARD interactions between NLRP1^CARD^ and ASC^CARD^.

Size-exclusion chromatography was used to test the effects of the mutants MBP-ASC^CARD^ D143A/E144A (MBP-ASC^CARD-DE^) and MBP-ASC^CARD^ N128A/E130R (MBP-ASC^CARD-NE^) on the MBP-ASC^CARD^/NLRP1^CARD^ complex formation in solution. MBP-ASC^CARD-DE^ and MBP-ASC^CARD-NE^ failed to form supramolecular complexes with NLRP1^CARD^ (Fig. 3h, i). These findings showed that D143, E144, N128, and E130 on the ASC^CARD^ play an important role in mediating the interaction between NLRP1^CARD^ and ASC^CARD^.

### The CARD–CARD interactions between NLRP1 and ASC utilize three asymmetric interaction types of death-fold superfamily

**Fig. 4:**
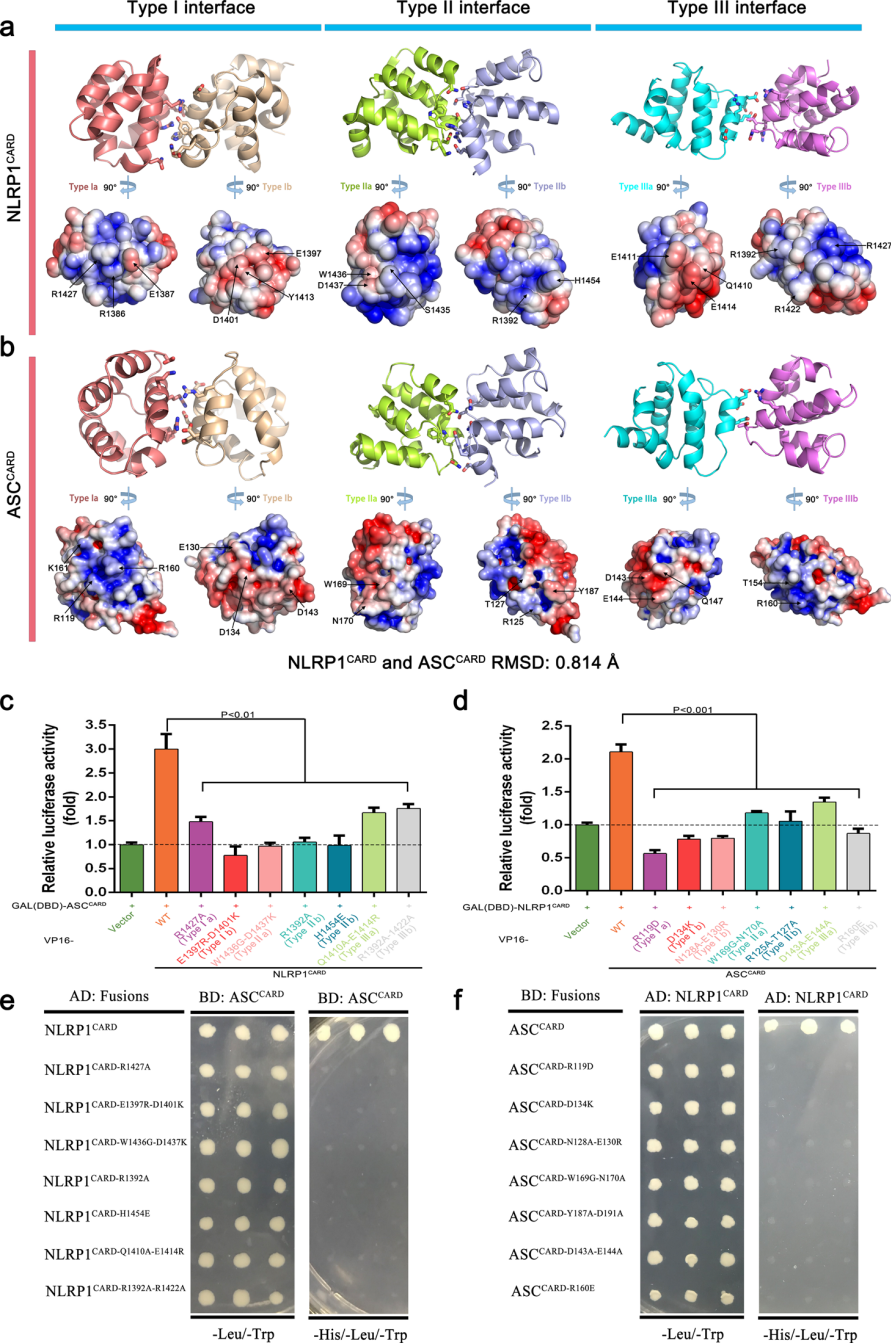
NLRP1^CARD^ interacts with ASC^CARD^ by three asymmetric interaction types of DD superfamily. **a** Model for Type I (deepsalmon and wheat), II (limon and lightblue), and III (cyan and violet) interface residues and electrostatic surface of NLRP1^CARD^ generated from the ASC^CARD^ structure as template (PDB 6N1H). The key residues are shown and each of the colors represents the same interface type. **b** Structure for Type I (deepsalmon and wheat), II (limon and lightblue), and III (cyan and violet) interface residues and electrostatic surface of ASC^CARD^ (PDB 6N1H). The key residues are shown and each of the colors represents the same interface type. **c**, **e** Measurement of the interaction between wild-type ASC^CARD^ and NLRP1^CARD^ with different mutations by the M2H and Y2H experiments. Luciferase activity in the HEK293T cells was normalized to *Renilla*. The data were presented as the fold of negative control in **c** and (−) denotes empty vector control in **e**. Mean values ± SEM are representative of three independent experiments. **d**, **f** Measurement of the interaction between wild-type NLRP1^CARD^ and ASC^CARD^ with different mutations by the M2H and Y2H experiments. Luciferase activity in the HEK293T cells was normalized to *Renilla*. The data were presented as the fold of negative control in **d** and (−) denotes empty vector control in **f**. Mean values ± SEM are representative of three independent experiments.

As with the prior reports, some members of the DD superfamily utilize three distinct interface types to assemble macromolecular complex structure^11,31,37^.The major interacting amino acids at the AI interface of NLRP1^CARD^ are part of the three asymmetric interaction types. R1427 is at the Type Ia interface of NLRP1^CARD^ and the residues R1392 and R1422 are at Type IIIb interface of NLRP1^CARD^. The N128 and E130 residues at the Type Ib interface on ASC^CARD^ and the residues D143 and E144 at the Type IIIa interface on ASC^CARD^ play an important role in mediating the interaction between NLRP1^CARD^ with ASC^CARD^^22,31^. The percentage of NLRP1^CARD^ in the MBP-ASC^CARD^/NLRP1^CARD^ complex was higher than in other DD proteins when binding with ASC^CARD^ (Fig. 1a and Supplementary Fig. S1a). To our surprise, NLRP1^CARD^ possesses a structure conformation similar to the conformation of human ASC^CARD^ (RMSD 0.814 Å), perhaps indicating that NLRP1^CARD^ and ASC^CARD^ may be better interactors (Fig. 4a, b). In addition, PISA analysis of predicted buried surface area in NLRP1^CARD^ and ASC^CARD^ showed that the total buried interface area is larger than a CARD-containing molecular CARD9^CARD^ interacts with ASC^CARD^ or NLRP1^CARD^ (Supplementary Table S2). This evidence suggests us that NLRP1^CARD^ may co-assemble with ASC^CARD^ to form polymeric filaments.

We also investigated whether mutations in the three asymmetric interfaces could interfere with the interaction between NLRP1^CARD^ and ASC^CARD^ to test the hypothesis that NLRP1^CARD^ could co-assemble with ASC^CARD^ to form polymeric filaments. Examination of the electrostatic charge surface of the NLRP1^CARD^ and ASC^CARD^ structures revealed that Type I and III interfaces contain patches of surface charges explaining the charge complementarity of the two interaction types (Figs. 1c, d and 4a, b). However, the Type II interface of the NLRP1^CARD^ and ASC^CARD^ has both ionic and hydrophobic interactions (Fig. 4a, b). The similarity of the electrostatic charge surface suggests that the NLRP1^CARD^ may be more suitable to form filaments with ASC^CARD^ (Fig. 4a, b). R1427 and D1401 on the NLRP1^CARD^ Type Ia and Ib surface are similar to the corresponding position R160 and D134 in ASC^CARD^, respectively. We generated a series of mutations at the Type I, II and III interfaces of NLRP1^CARD^ and ASC^CARD^, investigating the interaction between NLRP1^CARD^ and ASC^CARD^ in M2H and Y2H assays. We found that every mutation in the interface of NLRP1^CARD^ and ASC^CARD^ decreased NLRP1^CARD^ and ASC^CARD^ interaction, supporting the NLRP1^CARD^ and ASC^CARD^ filament formation mechanism (Fig. 4c–f). The results on the interaction between NLRP1^CARD^ and ASC^CARD^ showed that their interaction and their copolymerization into filaments depend on the three asymmetric interaction types of the DD superfamily.

### Three asymmetric interaction types of NLRP1^CARD^ and ASC^CARD^ are verified with key functional roles in NLRP1 inflammasome

**Fig. 5:**
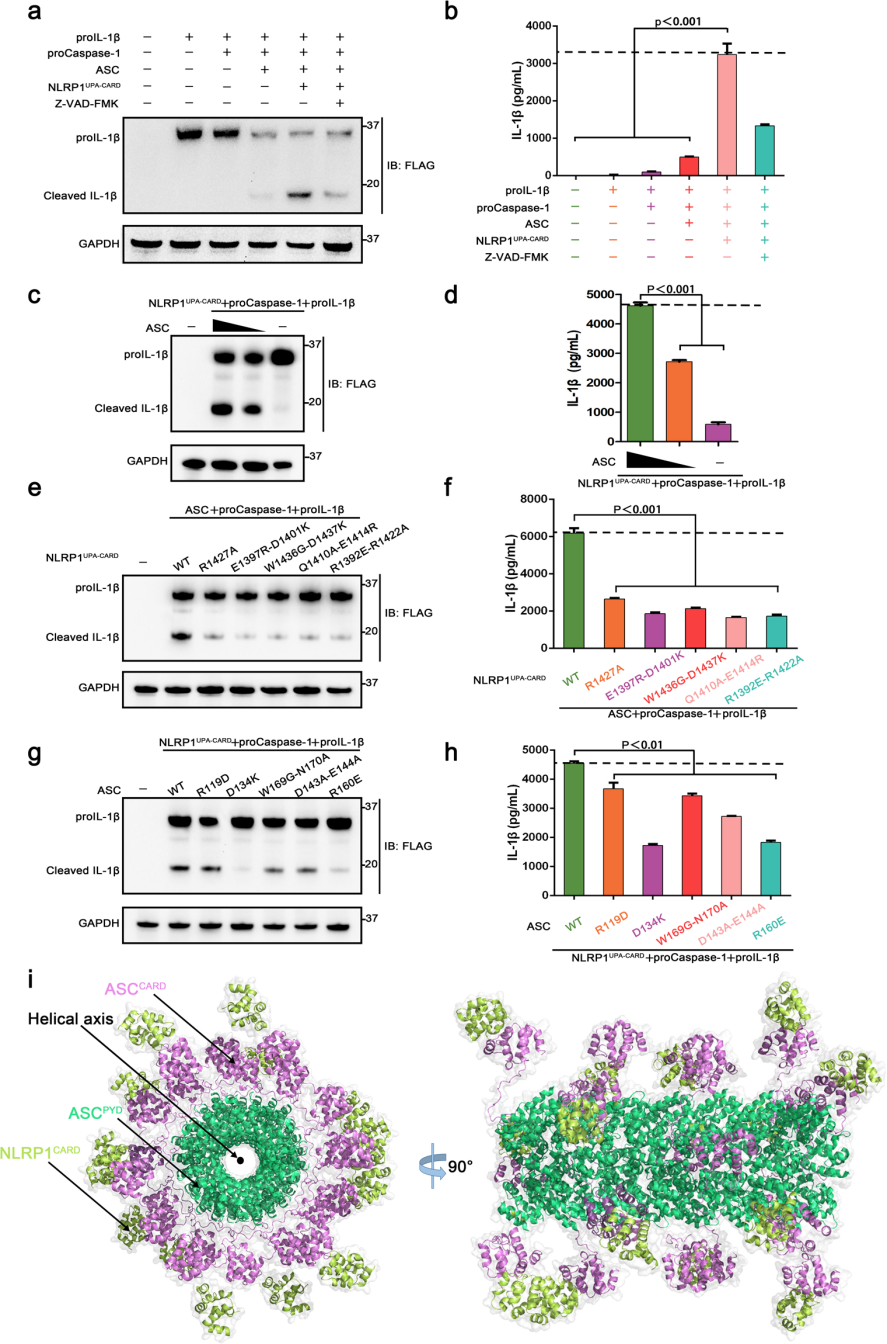
Three asymmetric interaction types of CARD are verified with key functional roles in NLRP1 inflammasome activation. **a**, **b** Reconstitution of NLRP1 inflammasome in HEK293T cells. Inflammasome was activated by co-expression of the UPA-CARD fragment of NLRP1 and was monitored by immunoblot (IB) or ELISA for cleaved IL-1β. Mean values ± SEM are representative of three independent experiments. **c**, **d** Western blotting and ELISA analysis for cleaved IL-1β from ASC-deficient NLRP1 inflammasome. Mean values ± SEM are representative of three independent experiments. **e**, **f** Western blotting and ELISA analysis for cleaved IL-1β from wild-type NLRP1 and NLRP1 mutations in NLRP1 inflammasome. Mean values ± SEM are representative of three independent experiments. **g**, **h** Western blotting and ELISA analysis for cleaved IL-1β from wild-type ASC and ASC mutations in NLRP1 inflammasome. Mean values ± SEM are representative of three independent experiments. **i** Structure model for the filament co-assembly of NLRP1^CARD^ and ASC in the complex. The top view is shown schematically at the left and the side view is shown schematically at the right.

The cleavage of pro-interleukin-1β (IL-1β) is a major marker of NLRP1 inflammasome activation. The NLRP1 autoproteolytic fragment (UPA-CARD), but not the only CARD domain, mediates NLRP1 inflammasome constitutive activation in HEK293T cells^17,38^. To study if the three asymmetric interaction types of DD superfamily affect the NLRP1 inflammasome activation, we reconstituted the NLRP1 inflammasome in HEK293T cells. NLRP1^UPA-CARD^, ASC, pro-caspase-1, and pro-IL-1β expression plasmids were co-transfected into HEK293T cells. When pro-IL-1β alone was overexpressed, mature IL-1β was undetectable, indicating the lack of inflammasome activation in HEK293T cells. Co-expression of NLRP1^UPA-CARD^ produced the cleavage products of pro-IL-1β, demonstrating the successful reconstitution of NLRP1 inflammasome (Fig. 5a, b). The increased secretion of mature IL-1β was blocked by the small molecule cysteine protease inhibitors Z-VAD-FMK. These observations indicate that NLRP1^UPA-CARD^ contributes to NLRP1 inflammasome activation, pro-IL-1β maturation and secretion in HEK293T cells. As ASC has been shown to be dispensable in murine NLRP1B inflammasome activation^15,39^, we characterized the function of ASC in human NLRP1 inflammasome activation in HEK293T cells. We altered the expression of ASC in HEK293T cells, showing that ASC could increase the inflammasome activation levels (Fig. 5c, d). These results showed that ASC functions as an indispensable adaptor to promote the human NLRP1 inflammasome activation. Altogether, these results indicated that a functional NLRP1 inflammasome is established in HEK293T cells and that ASC is an indispensable adaptor in the assembly of human NLRP1 inflammasome.

To ascertain the roles of the three asymmetric interaction types of NLRP1^CARD^ and ASC^CARD^ in NLRP1 inflammasome assembly, we performed systematic site-directed mutagenesis in the reconstituted HEK293T system. Co-expression of NLRP1^UPA-CARD^ mutants, ASC, pro-caspase-1, and pro-IL-1β caused a significantly decreased processing of pro-IL-1β by caspase-1 (Fig. 5e, f). These results established that the three asymmetric interaction types of NLRP1^CARD^ mediate NLRP1 inflammasome activation. Mutations in ASC, which is a dispensable adaptor in murine NLRP1B inflammasome, could lead to decreased maturation and secretion levels of IL-1β (Fig. 5g, h). The C-terminal fragment of NLRP1 co-assembled with ASC to form a functional inflammasome complex to amplify danger signals in the activation mechanism of NLRP1 inflammasome, which is consistent with the Mosaic model (Fig. 5i). The functional characterization of NLRP1^CARD^ and ASC^CARD^ confirmed that all three asymmetric interaction types in NLRP1^CARD^ and ASC^CARD^ participate in danger signal sensing and signal transduction amplification in the NLRP1 inflammasome.

## Discussion

The C-terminal fragment of NLRP1 recruits ASC to amplify signal transduction through CARD–CARD instead of PYD–PYD homotypic interactions in ASC-dependent NLRP1 inflammasome^15,17,27^. Our findings established that a mosaic model of NLRP1^CARD^ and ASC^CARD^ molecular interaction details. After auto-processing of FIIND domain, the released C-terminal fragment increases the local concentration of ASC in heteromeric and homomeric interactions so that the C-terminal fragment of NLRP1 and ASC can co-assemble into higher-order signalosomes and amplify information regarding receptor activation^36,40^. Formation of the molecular platform, activates effector molecule caspase-1, which then cleaves IL-1β, IL-18, and Gasdermin D, eventually leading to cell death.

NLRP1 and ASC are members of death-fold containing proteins. A recent study proposed that the death-fold containing inflammasome assembles with a unified nucleation-induced mechanism^11^. In this model, death-fold containing receptors, i.e., NLRP3 and AIM2, nucleate ASC filaments interactions for efficient activation and signal amplification^41^. In the AIM2^PYD^/ASC^PYD^ or NLRP3^NBD-PYD^/ASC^PYD^ filament complex, ASC^PYD^ forms the main filament body and the AIM2^PYD^ or NLRP3^NBD-PYD^ is localized at one end of the complex and exists very less percentage, but not in the NLRP1^CARD^/ASC^CARD^ filament. However, some of our results differ from the mechanisms that show copolymerization in filament formation from death-fold containing proteins such as ICEBERG–Caspase-1^CARD^ interactions and NLRP3^PYD^–ASC^PYD^ interactions^42,43^, which are similar to the NLRP1^CARD^/ASC^CARD^ interactions in our study. Previously, the truncation mutants of NLRP1 indirectly proved that NLRP1 interacts with ASC via homotypic CARD–CARD interactions in cells^23,44^. Our results provided direct evidence of the binding between purified NLRP1^CARD^ and ASC^CARD^ proteins in an in vitro proteins solution.

We focused on the molecular details of human NLRP1 inflammasome assembly and presented evidence for the mechanism of NLRP1^CARD^ and ASC^CARD^ interactions in the formation of archetypical supramolecular filament during inflammasome activation. We confirmed that the interaction of NLRP1^CARD^ and ASC^CARD^ is primarily composed of ionic interactions. We showed that the asymmetric CARD–CARD interfaces in the NLRP1^CARD^ crystals mediate the interaction between NLRP1^CARD^ and ASC^CARD^ through charge complementarity. We generated a high-resolution crystal structure of human ASC^CARD^ and discovered that CARD structures are highly conserved with a six-helical bundle with a Greek key topology. Structure-directed mutagenesis on ASC^CARD^ demonstrated that the three interaction types of the DD superfamily play critical roles in ASC^CARD^ oligomerization. Finally, we showed that the primary structure sequence, the secondary structure, and the three-dimensional structure of NLRP1^CARD^ and ASC^CARD^ are similar. The high similarity suggests the possibility that the NLRP1^CARD^ can assemble with ASC^CARD^ and form the signal transduction complexes, i.e., filamentous structures, using the three asymmetric interfaces of DD superfamily. Mutagenesis and functional studies of NLRP1^CARD^ and ASC^CARD^ of the three asymmetric interfaces of the DD superfamily further supported that the archetypical supramolecular filament of NLRP1^CARD^ co-assembles with ASC^CARD^ to serve as an inflammasome signal amplification platform in NLRP1 inflammasome. In summary, our study presents a clear picture of a mosaic model of ASC-dependent signal amplification in human NLRP1 inflammasome activation.

## Materials and methods

### Plasmids and reagents

For protein expression in *Escherichia coli*, PCR-amplified NLRP1^CARD^ encoding residues 1397–1462 (NM_033004.3), ASC^CARD^ encoding residues 95–195 (NM_013258.4) from a cDNA library, and mutants of NLRP1^CARD^ and ASC^CARD^ were ligated into a pET30a-derived vector with an N-terminal MBP tag, His-tag, and a Tobacco Etch Virus (TEV) protease site between the MBP tag and target protein. In the Y2H assays, pGAD-T7 with activation domain and pGBK-T7 with binding domain plasmids were used with SalI and NotI sites. The pBIND vector contains a DNA-binding domain and *Renilla reniformis* luciferase sequence, whereas the pACT vector contains the VP16 activation domain in the M2H system. The human NLRP1^UPA-CARD^ started at Ser1213; full-length of ASC, pro-Caspase-1, and IL-1β were inserted into a modified pcDNA3.1 vector. All site-directed mutants were constructed by overlapping PCR or QuikChange and verified by DNA sequencing. The following antibodies were used: anti-FLAG (F1804–50UG, Sigma), anti-His (D191001, Sangon Biotech), anti-Strep: (688202, Biolegend), anti-GAPDH (AC002, ABclonal), anti-β-actin (AC004, ABclonal), and Human IL-1β Enzyme-linked immunosorbent assay (ELISA) Kit (557953, BD Biosciences).

### Protein expression and purification

The purification of the members of the DD superfamily was described previously^45^. In brief, for large-scale protein expression, the plasmids were transformed into Rosetta^TM^ BL21 (DE3) strain and the bacterial cells were grown in a 37 °C shaker. When the OD_600_ of the cultures was ~1.2, the recombinant protein was induced overnight using isopropyl 1-thio-β-d-galactopyranoside at a final concentration of 0.3 mM at 16 °C. The bacterial cells were collected and lysed by sonication with buffer A (250 mM NaCl, 5 mM imidazole, 20 mM CHES-HCl pH 9.0) plus protease inhibitors (Roche, Basel, Switzerland). After centrifugation, the samples were purified on a Hisprep IMAC column (GE Healthcare) and eluted with high-concentrated buffer B (500 mM NaCl, 130 mM imidazole, 20 mM CHES-HCl pH 9.0). Then, the MBP tag was removed by adding TEV and 5 mM dithiothreitol (DTT) for overnight incubation. After using gel filtration to remove DTT and imidazole, the target protein was further purified with Hisprep IMAC columns (GE Healthcare) and a XK26/60 Superdex 200 size-exclusion column. The expression and purification of ASC^CARD^ were similar to that of NLRP1^CARD^, except that the MBP tag was not removed and 20 mM Tris-HCl pH 8.0 buffer was used. All mutated proteins were purified and expressed by the same methods as used for wild-type proteins.

### Crystallization

Before screening the crystallization conditions by setting up hanging drops, the recombinant MBP-ASC^CARD^ protein was concentrated to ~40 mg/mL. Crystallization conditions were tested with a series of commercial and home-designed crystallization kits. Two weeks later, the crystals of MBP-ASC^CARD^ appeared with conditions of 1.80 M ammonium sulfate and 0.1 M HEPES pH 7.0 at 18 °C. Crystals were flash cooled in cryoprotectant composed of additional 10% ethylene glycol, 20% sucrose, and 2% maltose (v/v) for X-ray diffraction data collection.

### X-ray diffraction, structure determination, and refinement

X-ray diffraction data collection was conducted at Advanced Photon Source beamline 23-ID-D and Shanghai Synchrotron Radiation Facility^46^. The data were processed with the HKL2000^47^ program suite and XDS^48^. For structure determination, molecular replacement was used for Phaser^49^ from the CCP4 program suite^50^ based on a MBP structure from the Protein Date Bank (code 3VD8). The original structure model was further improved by multiple rounds of model fitting with Coot^51^ and the structural refinement was conducted in the Phenix.refine^52^. The Molprobity server^53^ and RCSB ADIT validation server^54^ were used to validate the ASC^CARD^ structure model. Molecular graphics, structure superposition, and the calculation of RMSD between two structures were displayed by program Pymol (Schrodinger, LLC). Meanwhile, the electrostatic surface was calculated with PDB2PQR^55^ server and the electrostatic model was displayed with Pymol.

### Y2H assay

Based on the GAL4 Matchmaker, the Y2H system was used to examine the interaction of DD superfamily. The indicated genes were cloned into pGAD-T7 plasmid encoding activation domain and pGBK-T7 plasmid encoding DNA-binding domain. Combinations of pGBK-T7 and pGAD-T7 plasmid pairs were co-transformed to *Saccharomyces cerevisiae* strain AH109 cells following the manufacturer’s manual (Clontech, Mountain View, CA, USA). The yeast cells were first plated on agar plates without leucine and tryptophan (-Leu/-Trp) for selection and grown at 30 °C for 3 days. At least three separate positive colonies were picked and spotted on -Leu/-Trp and -His/-Leu/-Trp plates, and incubated for 3 days to determine positive interactions. The empty plasmids were also co-transformed into the AH109 cells as a negative control.

### Negative-stain EM

Briefly, NLRP1^CARD^ mixed with ASC^CARD^ physically for 4 h and the binary complex of NLRP1^CARD^/ASC^CARD^ was purified by size-exclusion chromatography at a final concentration of 0.2 μg/mL. The carbon coated 400-mesh Cu EM specimen grids (Solarus, Gatan, Model 950) were employed for incubating with 2.5 μL of sample for 90 s. Then, the grids were incubated for 90 s with 20 μL of stain (2% Uranyl Acetate) three times. Finally, the grids were air dried for examination by electron microscopy.

### CD spectroscope

The secondary structure and thermal stability of NLRP1^CARD^ and mutants were analyzed by CD spectra. All of the protein samples data were recorded using Chirascan Spectrometer (Applied Photophysics, Leatherhead, UK) with phosphate-buffered saline (pH 7.5). The far-ultraviolet (UV) CD spectral data were collected from 190 to 260 nm with a 1 mm rectangular cell path length and 0.5 mg/mL protein concentration at 20 °C. Thermal denaturation analysis was measured from 25 °C to 95 °C with data scanning from 190 to 260 nm at every 5 °C. With Boltzmann sigmoidal in Prism, the CD signals at 222 nm against temperature were used to calculate the *T*_m_, which evaluates the thermostability of NLRP1^CARD^ and mutated proteins of NLRP1^CARD^.

### MALS analysis

After the Superdex 200 10/30 size-exclusion column was equilibrated with buffer (500 mM NaCl, 20 mM CHES-HCl pH 9.0), 500 µL of the samples at 0.5 mg/mL concentration were injected onto the mini-DAWN Tristar (Wyatt Technologies, USA) to analyze the molecular mass and oligomeric state of NLRP1^CARD^ and mutants. Each protein at a flow rate of 0.5 mL/min was passed through UV detector, a refractometer, and a multi-angle laser light-scattering detector. The sample data were processed with the manufacturer’s software named ASTRA (Wyatt Tech). Relative weight-averaged molecular masses of samples were based on absorption coefficients from the calculated result of polypeptide sequence of proteins.

### M2H assay

With the Checkmate M2H system (Promega, Madison, WI, USA), the interactions between NLRP1^CARD^ and ASC^CARD^ including their mutations were investigated in the HEK293T cells as described previously^56^. Briefly, according to the instructions of the manufacturer, the indicated genes were inserted into pACT vector, which contains the VP16 activation domain and the DNA-binding domain of GAL4 vector (pBIND), respectively. These vectors were transiently co-transfected into HEK293T cells in the presence of pG5*luc* vector, which has the firefly luciferase reporter genes. Forty-eight hours later, HEK293T cells were collected and the firefly luciferase was determined by Dual Luciferase Reporter Gene Assay Kit (Beyotime). The empty pACT and pBIND vectors were co-transfected into cells as negative control. Data were measured for at least three independent experiments.

### In vitro NLRP1 inflammasome reconstitution assay

It is reported that HEK293T cells are often used to reconstitute inflammasome by co-transfection of all components because of its low expression level of inflammasome components^23,39,44^. The HEK293T cells were maintained in Dulbecco’s modified Eagle’s media supplemented with 10% fetal bovine serum, 100 U/mL penicillin, and 100 μg/mL streptomycin in a 37 °C and 5% CO_2_ incubator. Approximately 1 × 10^5^ HEK293T cells were seeded into 24-well plates for overnight incubation before transfection. The human NLRP1^UPA-CARD^ or NLRP1 mutations (172 ng), human ASC or ASC mutations (172 ng), human pro-Caspase-1 (86 ng), and human IL-1β (172 ng) were transfected into each well of the 24-well plates by lipo6000^TM^ according to the manufacturer’s instructions. About 26 h later, the level of secreted mature IL-1β was detected using ELISA kits and the HEK293T cells were collected and analyzed for western blotting.

### Western blotting and ELISA assay

About 26 h after transfection, the supernatants were collected and analyzed by ELISA kits (BD Biosciences) as per the manufacturer’s guidelines. The HEK293T cells were lysed with lysis buffer (20 mM Tris-HCl pH 7.5, 100 mM KCl, 5 mM MgCl_2_, and 0.3% NP-40) and kept on ice for 30 min. Then, the HEK293T cell samples were loaded onto SDS-PAGE gel alongside with a molecular weight marker. After transferring, the membranes were incubated with a 5% skim milk in Tris-buffer saline-0.1% Tween and analyzed for the target proteins by immunoblotting using mouse anti-His, anti-Strep, anti-β-actin, and anti-GAPDH antibodies, respectively. The positive bands were developed with an ECL kit and visualized with Bio-Rad Gel Doc System.

### Statistical analysis

Data analysis was conducted with GraphPad Prism 5.0 software. All data of at least three independent experiments are representative of SD or mean and SEM. *P*-values < 0.05 were considered as statistically significant.

### Accession number

Atomic coordinates and structural factors of MBP-ASC^CARD^ have been deposited in the Protein Data Bank (https://www.rcsb.org/) and the accession code is 6KI0.

## Supplementary information

Figure S1

Figure S2

Figure S3

Figure S4

Figure S5

Table. S1

Table S2.

Supplementary information Figures legent
