# Supplementary figures and images for: Homotypic CARD-CARD interaction is critical for the activation of NLRP1 inflammasome
Source: Cell Death Dis. 2021 Jan 11;12(1):57. doi: 10.1038/s41419-020-03342-8 (PMC7801473; doi:10.1038/s41419-020-03342-8)

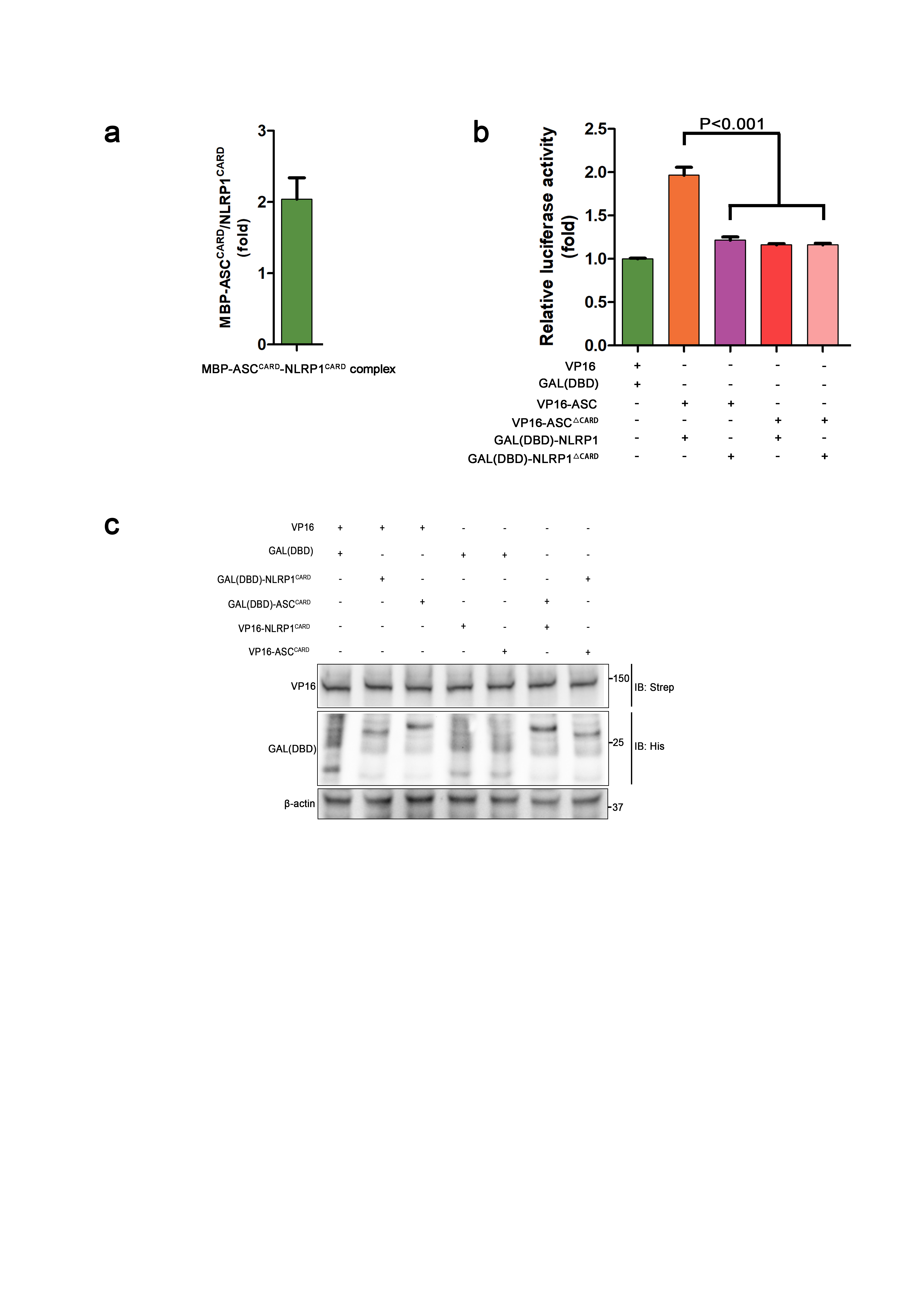

Supplement: Supplementary file 1 — Figure S1 [file 41419_2020_3342_MOESM1_ESM.png]

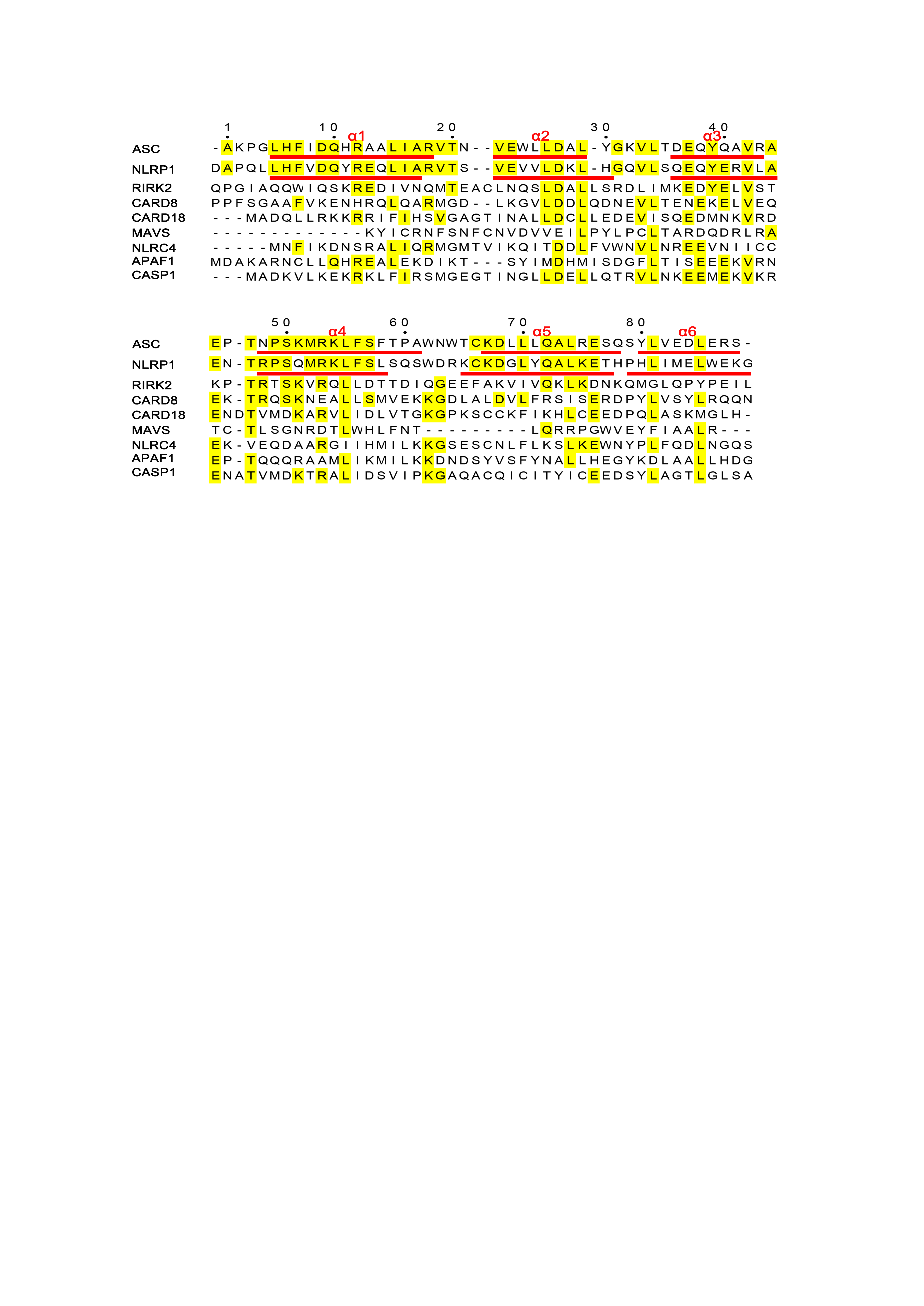

Supplement: Supplementary file 2 — Figure S2 [file 41419_2020_3342_MOESM2_ESM.png]

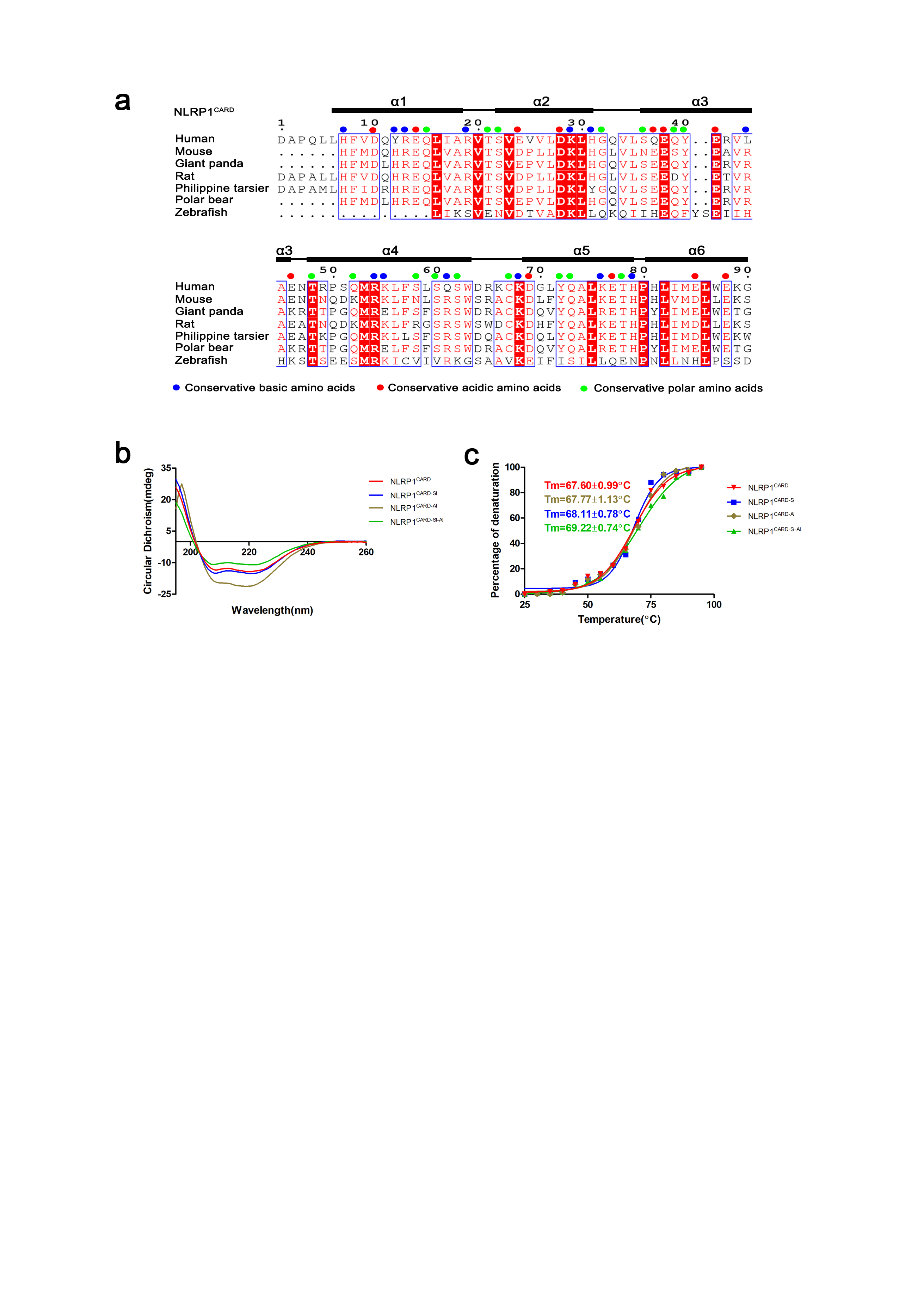

Supplement: Supplementary file 3 — Figure S3 [file 41419_2020_3342_MOESM3_ESM.png]

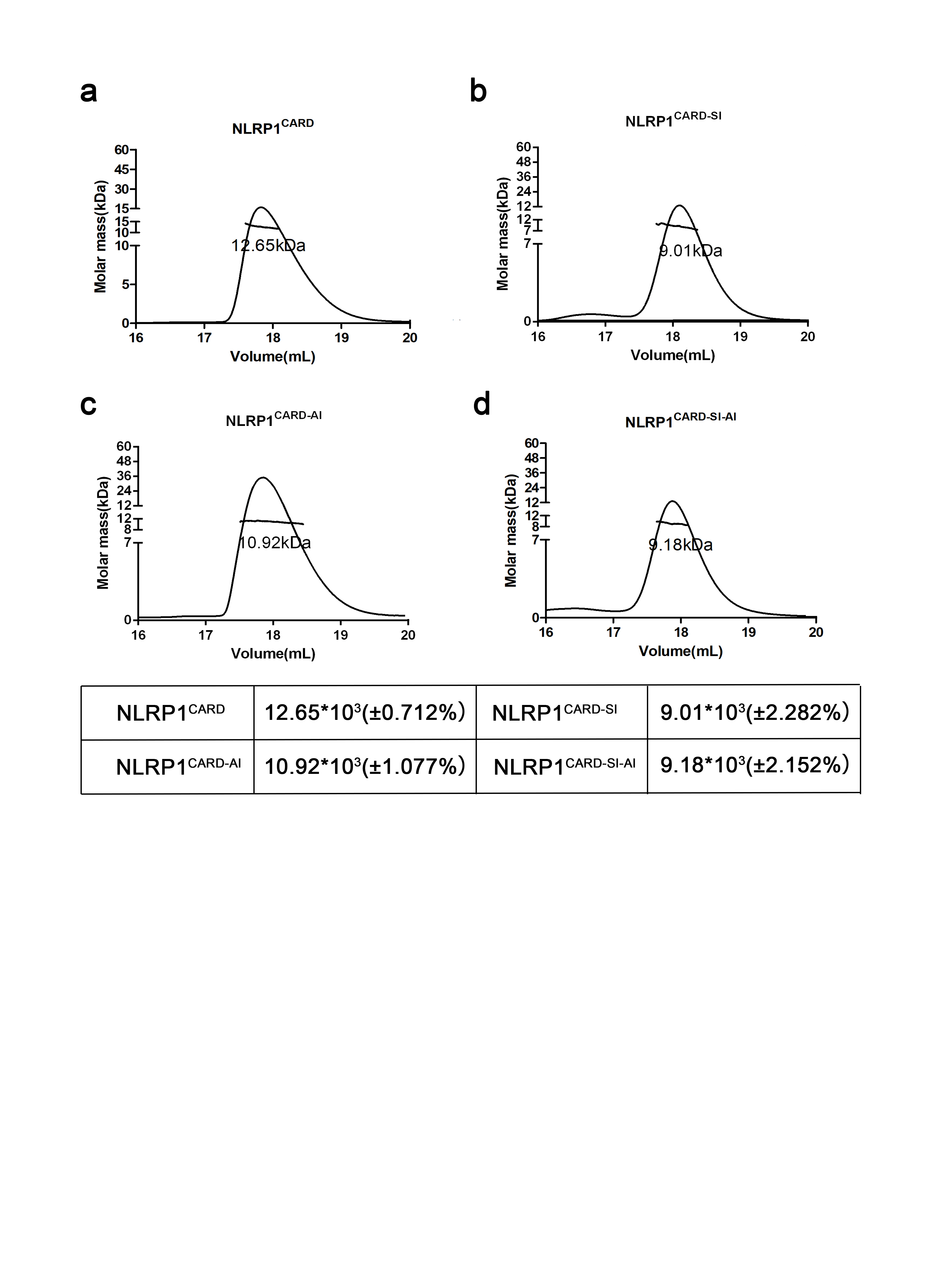

Supplement: Supplementary file 4 — Figure S4 [file 41419_2020_3342_MOESM4_ESM.png]

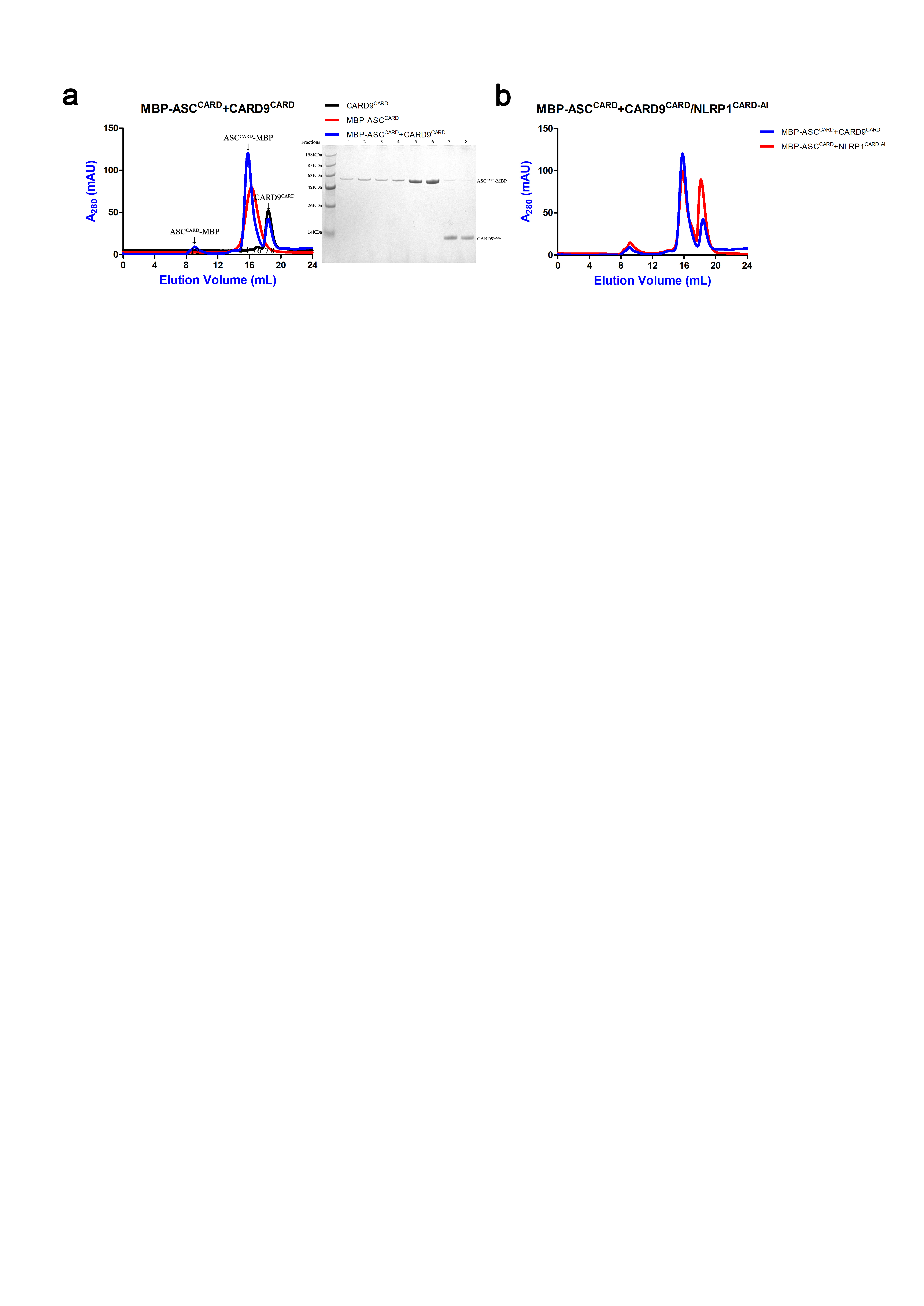

Supplement: Supplementary file 5 — Figure S5 [file 41419_2020_3342_MOESM5_ESM.png]
