# Supplementary material for: Homotypic CARD-CARD interaction is critical for the activation of NLRP1 inflammasome
Source: Cell Death Dis. 2021 Jan 11;12(1):57. doi: 10.1038/s41419-020-03342-8 (PMC7801473; doi:10.1038/s41419-020-03342-8)
Supplement: Supplementary file 6 — Table. S1 [file 41419_2020_3342_MOESM6_ESM.docx]

**Table. S1** X-ray data collection and refinement table of MBP-ASC^CARD^.

|  | MBP-ASC^CARD^ |
| --- | --- |
| Space group | C121 |
| Unit cell (a, b, c) (Å) | 175.76,56.02,146.93 |
| (α,β,γ) (°) | 90, 106.575, 90 |
| Resolution (Å) | 50-2.00 (2.12-2.00) ^*^ |
| No. of measured reflections | 346662(55798) ^*^ |
| No. of unique reflections | 92520(14772) ^*^ |
| Redundancy | 3.75 (3.78) ^*^ |
| Completeness (%) | 99.2 (99.0) ^*^ |
| *I/σ(I)* | 12.21 (1.76) ^*^ |
| *R_meas_* (%)^¶^ | 8.2 (129.2) ^*^ |
| *CC_1/2_* (%) | 99.8 (70.3) ^*^ |
| Refinement |  |
| Resolution (Å) | 50-2.00 |
| No. of resolution(work/test) | 2806/148 |
| No. of protein atoms | 7127 |
| No. of solvent/hetero-atoms | 269 |
| *B* factors (Å^2^) |  |
| Protein | 57.90 |
| Solvent | 46.82 |
| RMSD bond lengths (Å) | 0.008 |
| RMSD bond angles (°) | 1.23 |
| *R_work_* (%)^†^ | 22.03 |
| *R_free_* (%)^‡^ | 25.15 |
| Clash score | 5.51 |
| Overall MolProbity score | 1.34 |
| Ramachandran plot (favored/disallowed) ^**^ | 97.70/0 |
| PDB code | 6KI0 |

^*^Numbers with asterisk correspond to the last resolution shell.

^¶^ *R_meas_* = Σ_h_(n/n-1)^1/2^ Σ_i_ |I*_i_*(*h*) -<I(*h*)> | / Σ_h_Σ_i_ I_i_(*h*), where I_i_(*h*) and <I(*h*)> are the ith and mean measurement of the intensity of reflection *h*.

^†^ *R_work_* = Σ_h_||*F*_obs_ (*h*)|-|*F*_calc_ (*h*)|| / Σ_h_|*F*_obs_ (*h*)|, where *F*_obs_ (*h*) and *F* _calc_ (*h*) are the observed and calculated structure factors, respectively. No I/σ cutoff was applied.

^‡^*R_free_* is the R value obtained for a test set of reflections consisting of a randomly selected 5% subset of the data set excluded from refinement.
