# Supplementary material for: Homotypic CARD-CARD interaction is critical for the activation of NLRP1 inflammasome
Source: Cell Death Dis. 2021 Jan 11;12(1):57. doi: 10.1038/s41419-020-03342-8 (PMC7801473; doi:10.1038/s41419-020-03342-8)
Supplement: Supplementary file 7 — Table S2. [file 41419_2020_3342_MOESM7_ESM.docx]

**Table S2. The interface area of NLRP1^CARD^, ASC^CARD^ and CARD9^CARD^ with different predicted interaction types.**

**Table. S2**: The surface areas were calculated between the Type Ⅰa, Ⅱa, and Ⅲa surfaces of NLRP1^CARD^ and the Type Ⅰb, Ⅱb, and Ⅲb surfaces of ASC^CARD^ compared to the areas between the Type Ⅰa, Ⅱa, and Ⅲa surfaces of CARD9^CARD^ and the Type Ⅰb, Ⅱb, and Ⅲb surfaces of ASC^CARD^ or NLRP1^CARD^ by PISA server.

| Interfaces | Surface area (Å^2^) | Interfaces | Surface area (Å^2^) | Interfaces | Surface area (Å^2^) |
| --- | --- | --- | --- | --- | --- |
| NLRP1 (Ⅰa): ASC (Ⅰb) | 417.0 | CARD9 (Ⅰa): ASC (Ⅰb) | 393.9 | CARD9 (Ⅰa): NLRP1 (Ⅰb) | 426.4 |
| NLRP1 (Ⅱa): ASC (Ⅱb) | 416.6 | CARD9 (Ⅱa): ASC (Ⅱb) | 83.8 | CARD9 (Ⅱa): NLRP1 (Ⅱb) | 143.9 |
| NLRP1 (Ⅲa): ASC (Ⅲb) | 220.6 | CARD9 (Ⅲa): ASC (Ⅲb) | 83.9 | CARD9 (Ⅲa): NLRP1 (Ⅲb) | 38.4 |
| Total | 1054.2 | Total | 561.6 | Total | 608.7 |
