# Supplementary material for: Homotypic CARD-CARD interaction is critical for the activation of NLRP1 inflammasome
Source: Cell Death Dis. 2021 Jan 11;12(1):57. doi: 10.1038/s41419-020-03342-8 (PMC7801473; doi:10.1038/s41419-020-03342-8)
Supplement: Supplementary file 8 — Supplementary information Figures legent [file 41419_2020_3342_MOESM8_ESM.docx]

**Supplementary Materials**

**Fig. S1** NLRP1 recruits ASC through CARD domains. **a** The ratio of MBP- NLRP1^CARD^ and ASC^CARD^ in the MBP-ASC^CARD^/NLRP1^CARD^ complex was quantified by gray scales analysis. **b** Measurement of the interaction between wild-type full length NLRP1 and ASC by the M2H experiment. Luciferase activity in the HEK293T cells was normalized to *Renilla* and data were presented as the fold of negative control. Mean values ± SEM are representative of three independent experiments. **c** Immunostaining of cell lysates for Strep and His tag showing the expression of NLRP1^CARD^ and ASC^CARD^ in the M2H experiment and β-actin was as the loading control.

**Fig. S2** Primary sequence of NLRP1^CARD^ and ASC^CARD^ is more conserved than other CARD-containing proteins. **a** Structure-based [sequence alignment](https://www.sciencedirect.com/topics/biochemistry-genetics-and-molecular-biology/sequence-alignment) of 9 CARDs with known structures. Amino acid position is indicated on the top. The secondary structure of ASC^CARD^ and NLRP1^CARD^ are labeled with red lines. GenBank accession numbers for CARDs: ASC (NP_037390.2); [NLRP1](https://www.sciencedirect.com/topics/biochemistry-genetics-and-molecular-biology/nlrp1) (NP_127497.1); RIRK2 (NP_003812.1); CARD8 (XP_006723167.1); CARD18 (NP_067546.1); MAVS (NP_065797.2); NLRC4 (NP_001186067.1); APAF1 (NP_863651.1); and CASP1 (001244047.1).

**Fig. S3** The wild-type and mutants of NLRP1^CARD^ have the similar properties. **a** Structure-based sequence analysis of the NLRP1^CARD^ protein. Amino acid position is indicated on the top. GenBank accession numbers for NLRP1^CARD^: Human (NP_127497.1); Mouse (NP_001155886.1); Giant panda (XP_011215410.2); Rat (NP_001139227.2); Philippine tarsier (XP_008060230.1); Polar bear (XP_008686272.1); and Zebrafish (XP_009297081.1). **b** Circular dichroism (CD) spectra analysis for the NLRP1^CARD^ (red, continuous line); NLRP1^CARD-SI^ (blue, continuous line); NLRP1^CARD-AI^ (lightorange, continuous line); and NLRP1^CARD-SI-AI^ (green, continuous line). **c** Thermal denaturation assay of the wild-type and mutants of NLRP1^CARD^. The temperature range is from 25 to 95℃ varied in steps of 5℃ in the experiment.

**Fig. S4** Untagged NLRP1^CARD^ is in the form of several oligomers. **a** Static light scattering (SLS) analysis for the wild-type of NLRP1^CARD^. **b** Static light scattering (SLS) analysis for the NLRP1^CARD-SI^. **c** Static light scattering (SLS) analysis for the NLRP1^CARD-AI^. **d** Static light scattering (SLS) analysis for the NLRP1^CARD-SI-AI^.

**Fig. S5** Specificity of NLRP1^CARD^ and ASC^CARD^ interactions. **a** Size-exclusion chromatograph of the mixture MBP-ASC^CARD^ and CARD9^CARD^. ASC^CARD^ was fused with an N-terminal His-tag and incubated with untagged CARD9^CARD^, which was first purified by Ni-affinity chromatography. The protein eluted in the less aggregated position on a Superdex^TM^ 24 gel filtration column. **b** Comparative analysis for size-exclusion chromatograph of the mixture MBP-ASC^CARD^/NLRP1^CARD-AI^ and MBP-ASC^CARD^/CARD9^CARD^.
